# Liver proteomic response to hypertriglyceridemia in human-apolipoprotein C-III transgenic mice at cellular and mitochondrial compartment levels

**DOI:** 10.1186/1476-511X-13-116

**Published:** 2014-07-21

**Authors:** Grégory Ehx, Stéphanie Gérin, Grégory Mathy, Fabrice Franck, Helena CF Oliveira, Anibal E Vercesi, Francis E Sluse

**Affiliations:** 1 Laboratory of Bioenergetics (B22), Department of Life Sciences, University of Liege, Boulevard du rectorat 27, 4000 Liege, Belgium; 2 Departamento de Fisiologia e Biofísica, Instituto de Biologia, Universidade Estadual de Campinas, 13083-887 Campinas, SP, Brazil; 3 Departamento de Patologia Clínica, Faculdade de Ciências Médicas, Universidade Estadual de Campinas (UNICAMP), CEP: 13083-887 Campinas, São Paulo, Brazil

## Abstract

**Background:**

Hypertriglyceridemia (HTG) is defined as a triglyceride (TG) plasma level exceeding 150 mg/dl and is tightly associated with atherosclerosis, metabolic syndrome, obesity, diabetes and acute pancreatitis. The present study was undertaken to investigate the mitochondrial, sub-mitochondrial and cellular proteomic impact of hypertriglyceridemia in the hepatocytes of hypertriglyceridemic transgenic mice (overexpressing the human apolipoproteinC-III).

**Methods:**

Quantitative proteomics (2D-DIGE) analysis was carried out on both “low-expressor” (LE) and “high-expressor” (HE) mice, respectively exhibiting moderate and severe HTG, to characterize the effect of the TG plasma level on the proteomic response.

**Results:**

The mitoproteome analysis has revealed a large-scale phenomenon in transgenic mice, i.e. a general down-regulation of matricial proteins and up-regulation of inner membrane proteins. These data also demonstrate that the magnitude of proteomic changes strongly depends on the TG plasma level. Our different analyses indicate that, in HE mice, the capacity of several metabolic pathways is altered to promote the availability of acetyl-CoA, glycerol-3-phosphate, ATP and NADPH for TG *de novo* biosynthesis. The up-regulation of several cytosolic ROS detoxifying enzymes has also been observed, suggesting that the cytoplasm of HTG mice is subjected to oxidative stress. Moreover, our results suggest that iron over-accumulation takes place in the cytosol of HE mice hepatocytes and may contribute to enhance oxidative stress and to promote cellular proliferation.

**Conclusions:**

These results indicate that the metabolic response to HTG in human apolipoprotein C-III overexpressing mice may support a high TG production rate and that the cytosol of hepatocytes is subjected to an important oxidative stress, probably as a result of FFA over-accumulation, iron overload and enhanced activity of some ROS-producing catabolic enzymes.

## Introduction

Hypertriglyceridemia (HTG) is defined as a plasmatic triglyceride (TG) concentration exceeding 150 mg/dl. It occurs either as a consequence of genetic disorders (primary HTG) or as a feature of other metabolic diseases such as obesity, metabolic syndrome and diabetes (secondary HTG) [1]. It is also a risk factor for coronary heart disease [1], nonalcoholic fatty liver disease [2] and pancreatitis [3].

Apolipoprotein C-III (ApoC-III) is a glycoprotein component of TG-rich lipoproteins (mainly very-low density lipoproteins, VLDLs) which is involved in the control of TG metabolism in human [4] and mouse [5]. ApoC-III and TG plasmatic concentrations correlate [6] so that disruption of *ApoC-III* gene results in hypotriglyceridemia [5]. *In vivo* and *in vitro* studies have established that the control of ApoC-III on TG metabolism is achieved through at least two mechanisms: (i) inhibition of lipoprotein lipase (LPL) activity [7,8] and (ii) inhibition of hepatic uptake of TG-rich remnants (TRLs) [9,10].

In 1990, Ito *et al.* published a study in which they describe a transgenic mouse expressing the human ApoC-III in hepatocytes and, to a lower extend, in intestinal cells [11]. This mouse, called HuApoC-III, presents a HTG proportional to the number of human *ApoC-III* gene copies integrated in the genome. This feature allows distinguishing “low-expressor” (mild HTG) and “high-expressor” (severe HTG) HuApoC-III mice. Both categories are characterized by larger and more numerous VLDLs, whereas only high-expressor presents increased TG synthesis and secretion rates by hepatocytes [12]. HuApoC-III mice exhibit normal glucose homeostasis [13], body weight [14], adiposity [15], pancreatic insulin secretion and peripheral insulin sensitivity [16]. Therefore this mouse model is a particularly desirable model to study HTG without any potential interactive factors such as insulin resistance or obesity [17].

So far, HuApoC-III mouse has been principally studied through functional assays. At the hepatocyte level, bioenergetic studies showed that mitochondria isolated from HuApoC-III mouse present increased resting (state IV) and normal phosphorylating (state III) respiratory rates [18]. The higher state IV has been attributed to a higher activity of the mitochondrial ATP-sensitive K^+^ channel (mitoK_ATP_), resulting in a futile K^+^ cycling across the inner mitochondrial membrane and to a mild uncoupling which does not affect phosphorylation yield in state III [14,19]. Interestingly, it was also shown that HuApoC-III mouse hepatocytes are subjected to a remarkable oxidative stress, which has been attributed to an intracellular lipid accumulation and a higher free fatty acid (FFA) catabolism [20]. It has been hypothesized that mitoK_ATP_ uncoupling activity is a mechanism contributing to limit this stress in mitochondria [17]. At the organismic level, other functional studies revealed that HuApoC-III mouse presents higher liver oxygen consumption and body metabolic rate. This may explain its normal body weight despite a higher food intake [14].

Currently, the metabolic changes occurring in HuApoC-III hepatocytes remain poorly understood. In order to study global molecular adaptations set up in response to an environmental (nutriment composition, pathology, etc.) or an endogenous disruption (gene inactivation or overexpression, transgene expression, etc.), comparative proteomics has been proven to be a powerful tool [21]. In 2005, our group published a study [22] in which the proteomic changes occurring in the *ob/ob* mouse model hepatocyte mitochondria were studied using the 2D-DIGE (two dimensional-differential in-gel electrophoresis) technology [23]. In this mouse, an inactivated form of leptin is produced, resulting in obesity, hepatic steatosis and numerous other disorders. This study allowed us to characterize and understand the proteomic response occurring in *ob/ob* mouse hepatocytes and to provide new insights about steatosis.

In the present work, we used 2D-DIGE to characterize the mitochondrial proteomic response of HuApoC-III mouse hepatocytes to hypertriglyceridemia. This study was performed on both low-expressor (LE) and high-expressor (HE) HuApoC-III mice in order to assess a possible dose response effect of TG plasmatic concentration on mitochondrial proteomic adaptations. We also performed a 2D-DIGE analysis of the whole hepatocyte proteome in order to integrate the results obtained at the mitochondrial level in the cellular context. These different analyses enabled to draw a global overview of the liver metabolic response to hypertriglyceridemia and to propose literature-based hypotheses attempting to rationalize our proteomic observations.

## Results

### 2D-DIGE comparative analysis of the whole mitochondrial proteome

Using 2D-DIGE [21,23], the mitoproteomes of control (WT, TG = 40 to 80 mg/dl), low-expressor (LE, TG = 200 to 400 mg/dl) and high-expressor (HE, TG = 800 to 1000 mg/dl) mouse hepatocytes have been compared in order to characterize the dependence of the mitochondrial metabolism upon the TG plasmatic concentrations.

A total of 1721 protein spots were detected in 2D-gels (Additional file 1: Figure S1 and Additional file 2). Among them 757 exhibited a statistically significant expression variation (ANOVA-1 and t-test ≤ 0.05) between WT and LE mice or WT and HE mice and were pointed out as “protein spots of interest” (PSOI). Among them, 554 PSOI could be identified by mass spectrometry and were classified according to their localization: 431 (77.8%) corresponded to mitochondrial proteins (Additional file 1: Figure S2A), out of which the major part (97.6%) were matrix- (56.1%) or inner membrane- (41.5%) localized (Additional file 1: Figure S2B).

**Figure 1:**
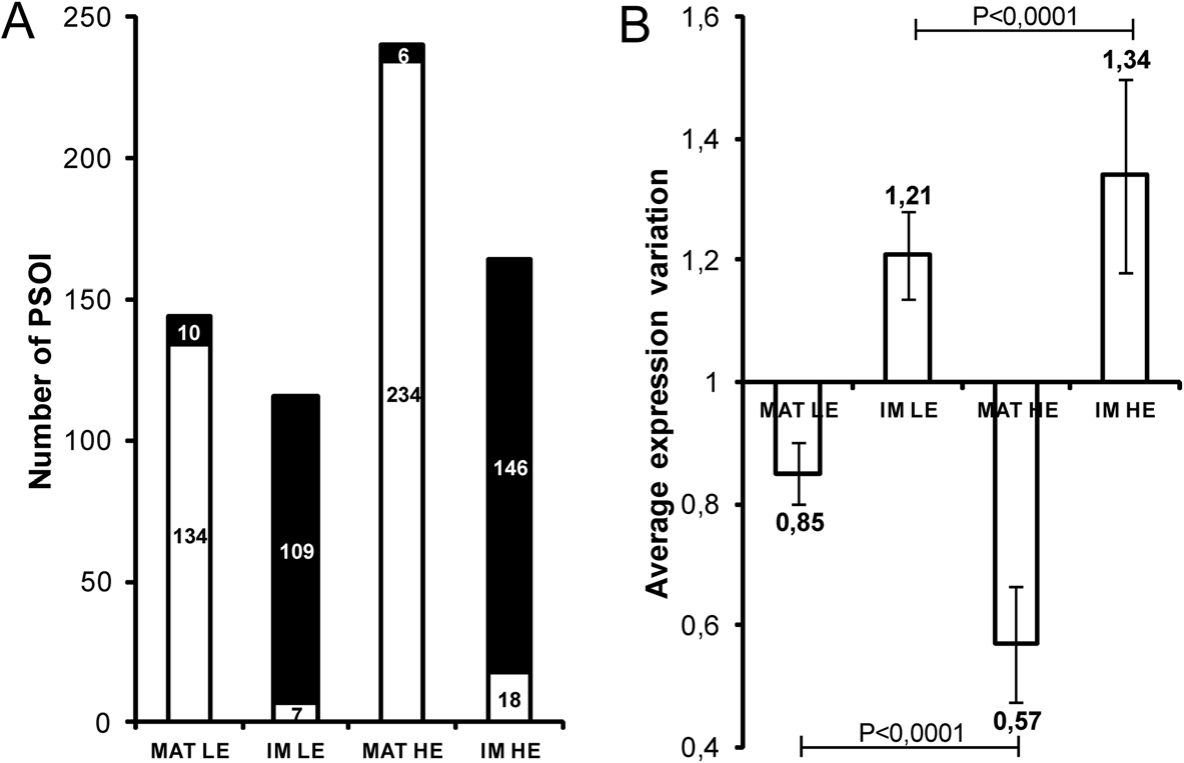
**Matricial and inner membrane PSOI expression variations in LE and HE mice compared to WT.** Panel **A** presents the number of down- (white) and up- (black) regulated PSOI. Panel **B** shows the average magnitude of PSOI expression variations. Matricial and inner membrane proteins were sorted out on the basis of localization data reported in UniProtKB/Swiss-Prot and KEGG Pathway. MAT, matrix; IM, inner membrane.

A total of 391 PSOI presented a statistically significant expression variation between WT and LE mice versus 716 between WT and HE mice. Out of these spots, 310 and 528 could be identified, respectively. Since matricial and inner membrane PSOI were the two major mitochondrial protein spot sub-populations of our analysis (Additional file 1: Figure S2B), we aimed to study the distribution of their expression variations for each proteomic comparison (LE or HE mice versus WT) (Figure 1). In Figure 1A, matricial and inner membrane PSOI have been classified according to the direction of their expression variation. Surprisingly, we noticed that most of the matricial and inner membrane PSOI are respectively down- (LE mice: 93.1%, HE mice: 97.5%) and up- (LE mice: 94.0%, HE mice: 89.0%) regulated in hypertriglyceridemic mice compared to WT. Figure 1B presents the average magnitude of matricial and inner membrane PSOI expression variations, showing that the amplitude of down- (matrix) or up- (inner membrane) regulations are more important in HE mice (matrix: 0.57, inner membrane: 1.34) compared to LE mice (matrix: 0.85, inner membrane: 1.21).Altogether, these results demonstrate that not only the number, but also the magnitude of mitochondrial proteomic changes strongly depends on the plasmatic TG concentration. Moreover, the general down- and up- regulation of the two major mitochondrial protein spot sub-populations (respectively matrix and inner membrane) (Figure 1A) tends to indicate that a large-scale phenomenon happens in hypertriglyceridemic mouse mitochondria. The relative proportion of matricial and inner membrane proteins is probably modified, leading to an increase of the (inner membrane)/(matricial) protein amount ratio. Since this phenomenon dramatically alters mitochondrial proteomic results by leading to an apparent down-regulation of matrix proteins and up-regulation of inner membrane proteins, it has the effect of preventing direct quantitation of individual protein spots expression variations inside each sub-mitochondrial compartment (e.g. an enzyme with unaltered abundance within the matrix proteome will appear down-regulated in mitochondrial comparison). In order to detect the potential adaptations of the mitochondrial pathways, both matrix and inner membrane proteomes had to be analyzed separately, as detailed in the next sections.

### Comparative analyses of sub-mitochondrial proteomes

#### Re-normalization of mitochondrial expression variations

**Figure 2:**
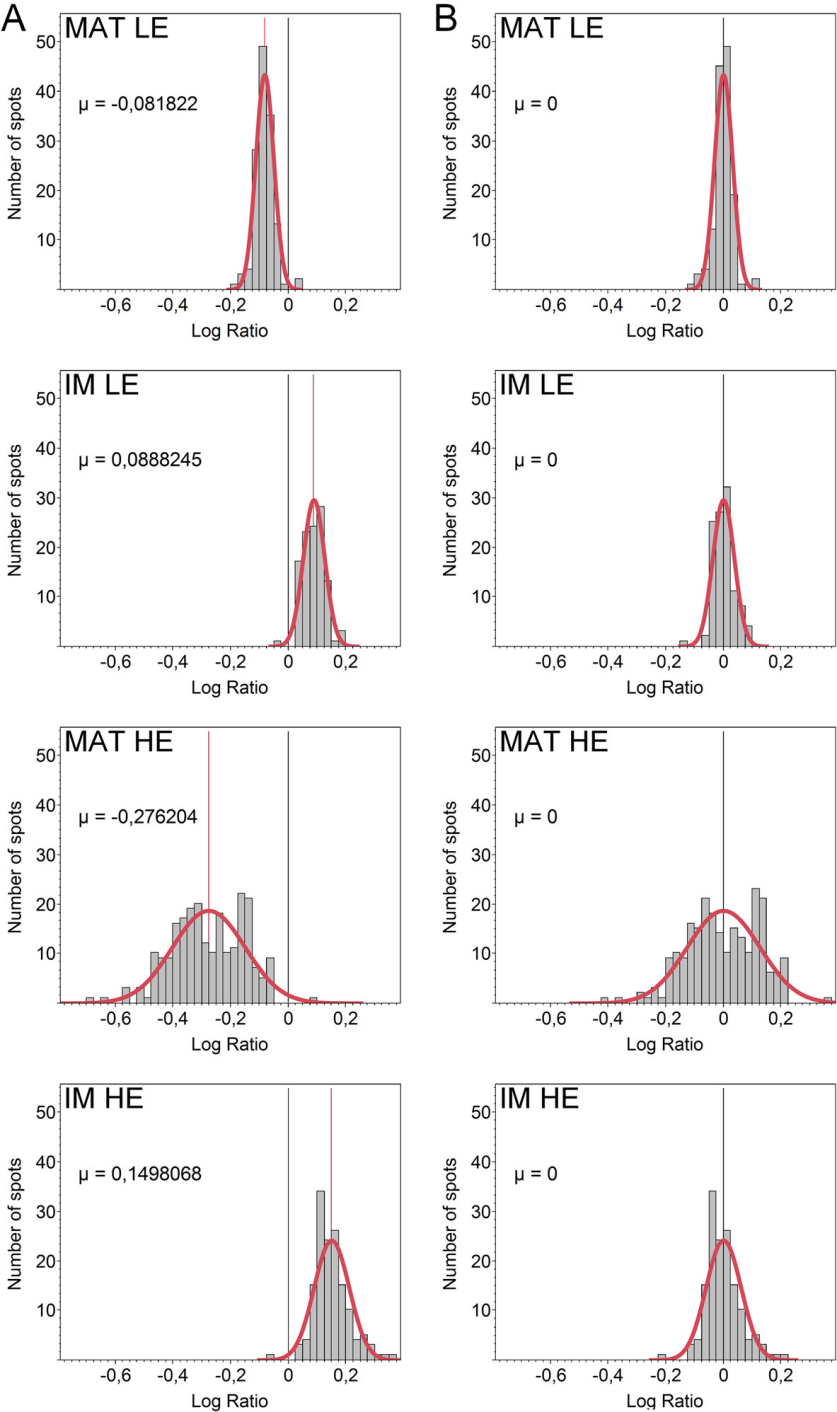
**Distribution charts of PSOI logarithmic expression variations obtained after excluding outliers (Additional files**3**and**4**).** In red, the normal adjustment curve and a straight line indicating the average of the distribution (μ). Panel **A**, before re-normalization; Panel **B**, after re-normalization. MAT, matrix; IM, inner membrane.

**Figure 3:**
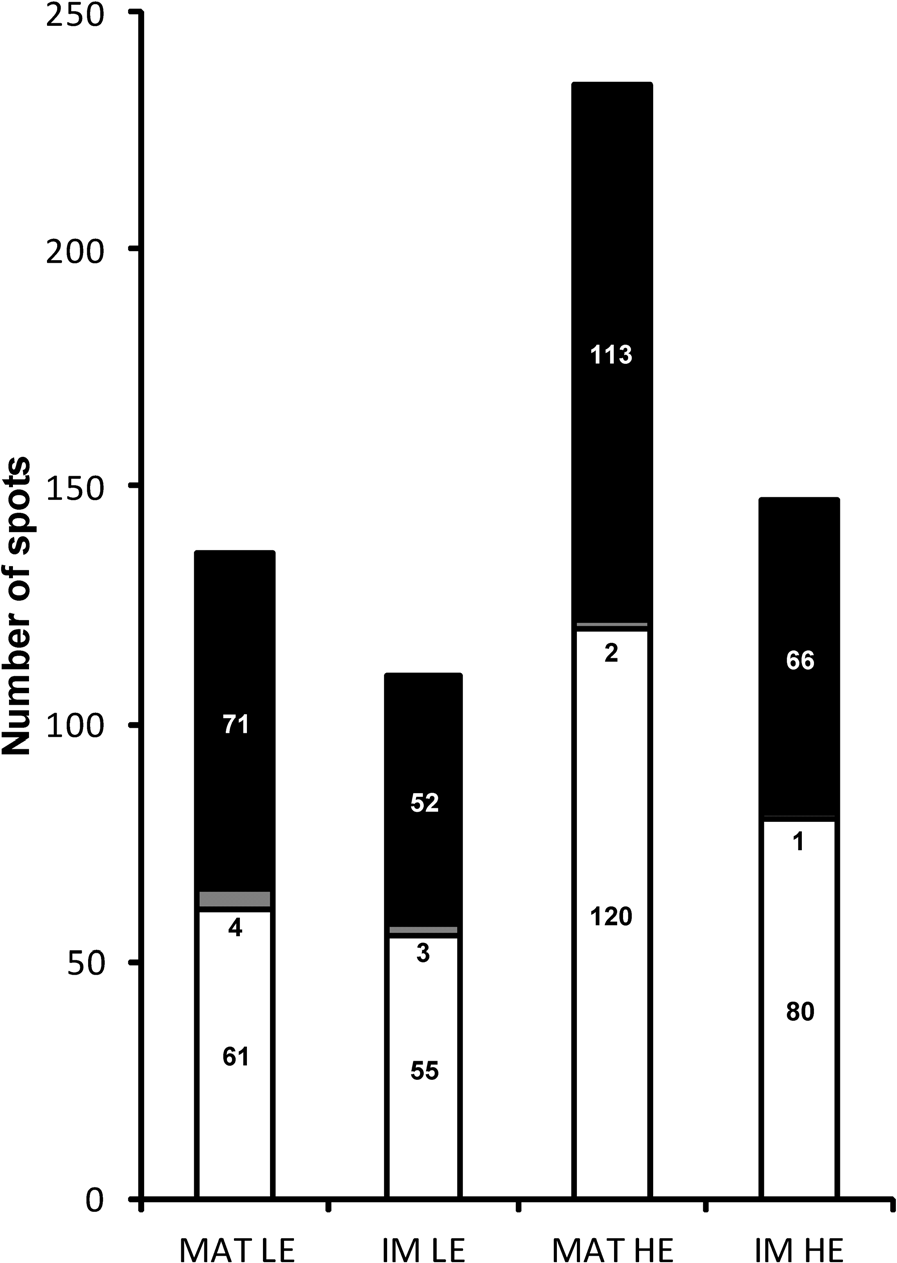
**Histogram classifying PSOI according to the direction of their re-normalized expression variation.** White, down-regulation; Black, up-regulation; Grey, absence of regulation. MAT, matrix; IM, inner membrane.

Since the 2D-DIGE analyses of matrix and inner membrane proteomes required performing 4 comparisons between experimentally isolated proteomes (MAT_WT_ vs MAT_LE_, IM_WT_ vs IM_LE_, MAT_WT_ vs MAT_HE_ and IM_WT_ vs IM_HE_), we first estimated the biological variation that would be observed in each of these comparisons. This estimation is based on the re-normalization of the results from the whole mitoproteome 2D-DIGE analysis (see Additional files 3 and 4 for detailed procedure description). In brief, we constructed the localization specific distribution charts (matrix and inner membrane) of PSOI logarithmic expression variations in each analysis (LE and HE mice). Then we re-centered these distributions on the abscissa axis origin by subtracting the average value of the distribution to the initial values (Figure 2). The resulting new logarithmic values were finally reconverted into non-logarithmic ones, corresponding to the re-normalized expression variations. In Figure 3, for LE and HE mice, the matricial and inner membrane re-normalized expression variations were classified according to their direction, showing that a similar proportion of PSOI (about 50/50) is down- and up- regulated in each case. Hence re-normalization makes it possible to estimate quantitative expression variations of protein spots inside each sub-mitochondrial compartment (matrix and inner membrane).

**Figure 4:**
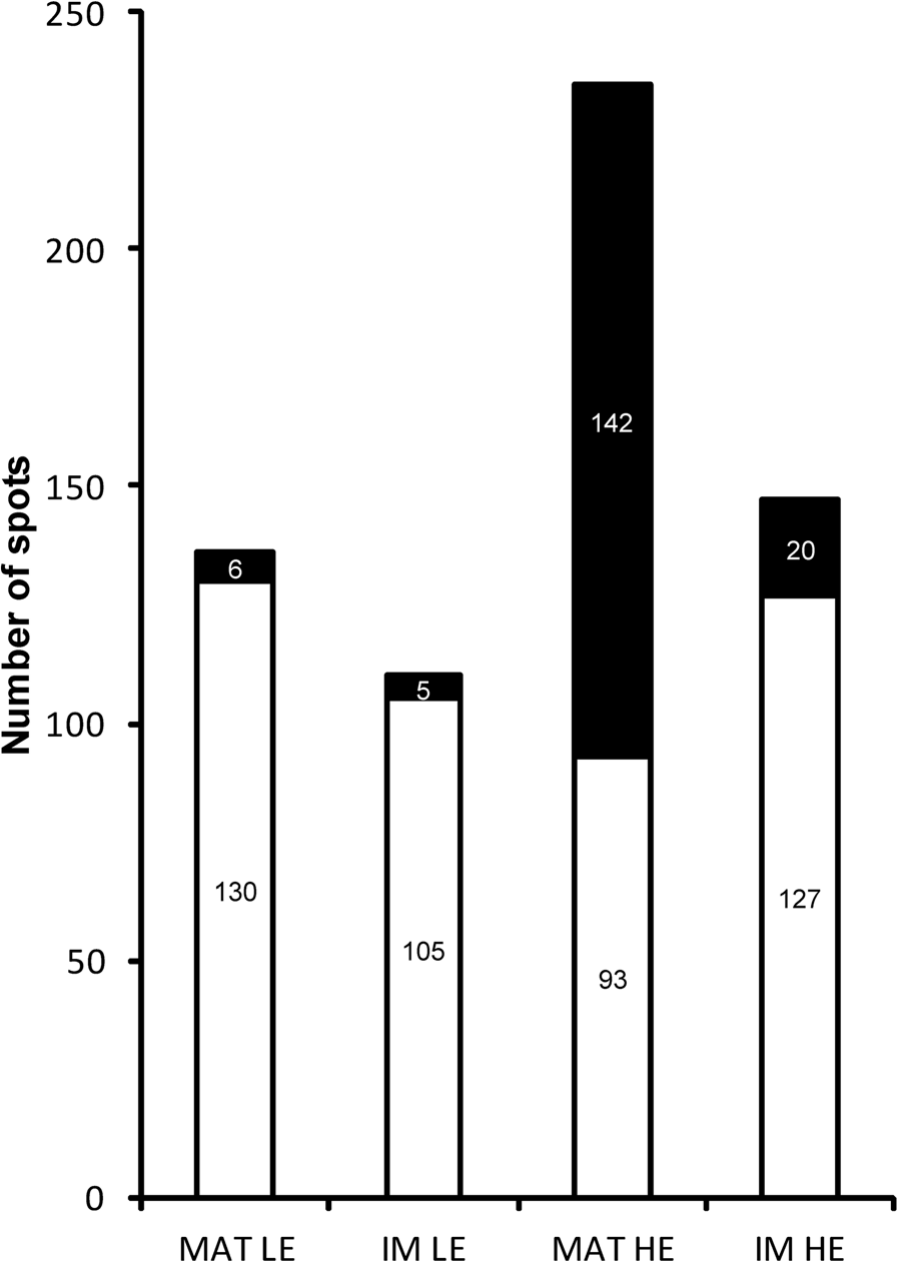
**Histogram illustrating the proportion of PSOI exhibiting a biologically relevant re-normalized expression variation (+/- 20%: ≤ 0.83 or ≥ 1.2).** Black, spots with a relevant expression variation; White, spots without a relevant expression variation. MAT, matrix; IM, inner membrane.

The re-normalized expression variations were considered as biologically relevant from +/-20% (≤ 0.83 or ≥ 1.2). Figure 4 presents the proportion of PSOI exhibiting a biologically relevant re-normalized expression variation. This figure indicates that the proteomic response is much more important in the HE mouse matricial proteome (60.4%) when it is compared to the three others (LE matrix: 4.4%, LE inner membrane: 4.5%, HE inner membrane: 13.6%). This observation is related to the wider variance of the normal distribution which characterizes the HE mouse matricial proteome (Additional file 1: Table S1 and Figure 2). This finding is particularly relevant if the number of PSOI exhibiting a biologically significant re-normalized expression variation is reported to the total number of protein spots (1721) which were initially detected in 2D-gels. In this case, their proportion is significant in the HE mouse matricial proteome (8.3%) and very low in the three others (LE matrix: 0.3%, LE inner membrane: 0.3%, HE inner membrane: 1.2%). Altogether, these results strongly suggest that a marked proteomic adaptation occurs within the mitochondrial matrix whereas the inner membrane proteome remains quite unchanged in case of severe hypertriglyceridemia. In case of moderate HTG, it seems that neither matrix nor inner membrane are subjected to a significant proteomic adaptation. A list of the PSOI included in the re-normalization processes is presented in Additional file 5 and summarizes the information developed in the present section.

#### Experimental 2D-DIGE analyses of sub-mitochondrial proteomes

Since the re-normalization of mitoproteome results predicted only a weak proteomic response in LE mice, 2D-DIGE sub-mitochondrial comparisons on experimentally isolated proteomes were only carried out between WT and HE mice.

##### Matricial proteome

2D-DIGE has been performed on experimentally isolated matrix proteome (see Materials and methods section). A total of 1307 protein spots were detected in 2D-gels (Additional file 1: Figure S3). Among them, 195 (14.9%) presented a statistically significant (t-test ≤ 0.01) and biologically relevant (+/- 20%: ≤ 0.83 or ≥ 1.2) expression variation, out of which 181 could be identified by mass spectrometry (Additional file 6). Among the identified spots, 173 (95.6%) correspond to matricial proteins.

##### Inner membrane proteome

A 2D-DIGE analysis was also carried out on the experimentally isolated inner membrane (see Materials and methods section for procedure description) proteome to confirm the absence of major metabolic adaptations in HE mice, as suggested by the re-normalization procedure. A total of 1433 protein spots were detected in 2D-gels (Additional file 1: Figure S4), out of which only 2 (0.1%) presented a statistically significant (t-test ≤ 0.01) and biologically relevant (+/- 20%: ≤ 0.83 or ≥ 1.2) expression variation. One of them could be identified as a very long-chain specific acyl-CoA dehydrogenase of the mitochondrial inner membrane (Additional file 7).

### Quantitative comparison of experimental results and re-normalization estimations

**Figure 5:**
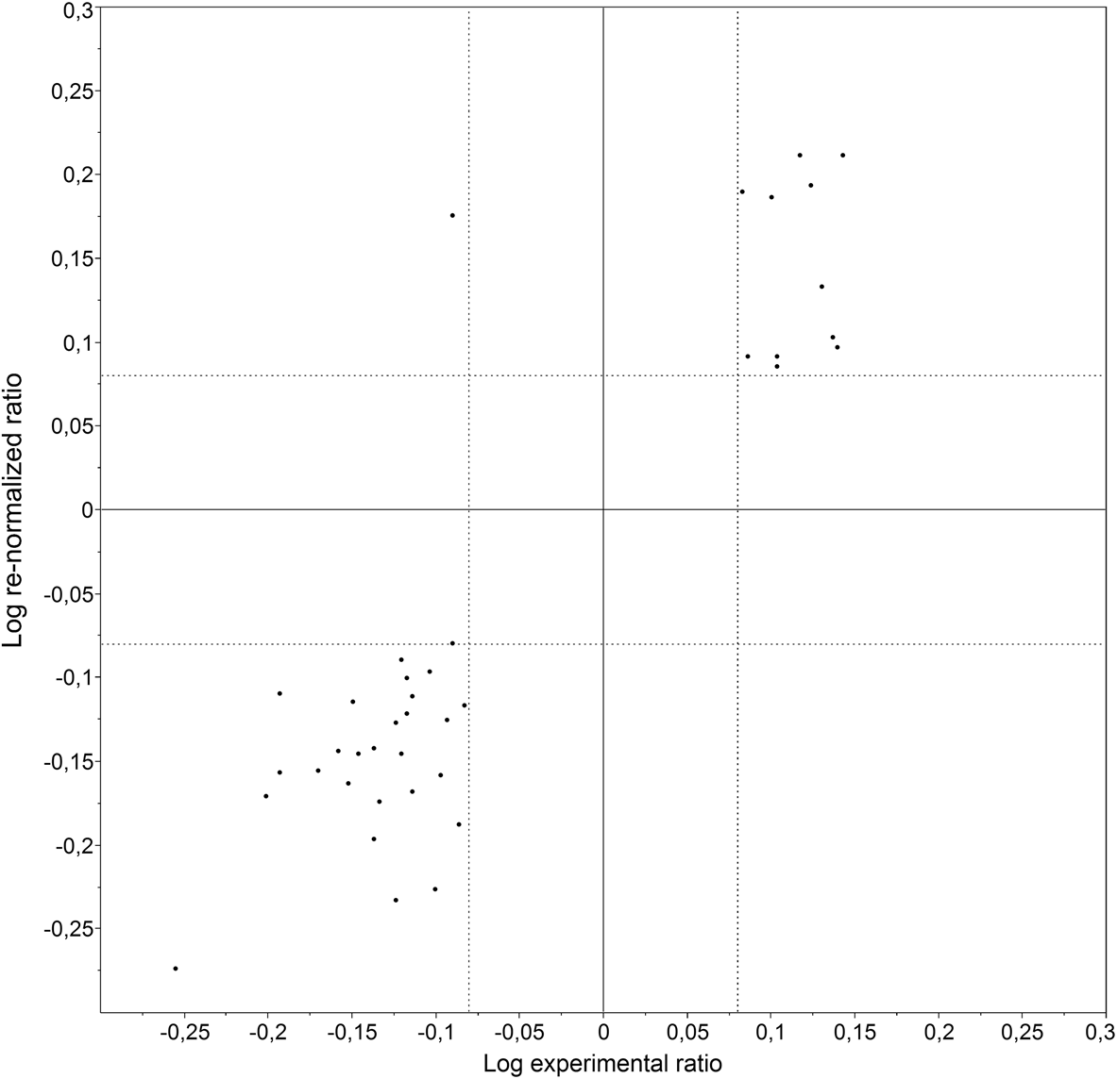
**Scatter plot comparing the expression variations obtained by re-normalization and experimental approaches.** Comparison has been performed for HE mouse matricial proteome (*x* axis: experimental approach; *y* axis, re-normalized values). Dotted lines indicate the biological significance cutoffs.

In order to demonstrate the accuracy of the re-normalization method, we intended to compare the experimental results and the estimations obtained by the re-normalization for the matricial proteome of HE mice. For this purpose, we only focused on the protein spots exhibiting a statistically significant and biologically relevant expression variation. We used DeCyder 7.0 to match these spots between the mitochondrial and matricial 2D-gels, allowing establishing a “spot-to-spot” correspondence on the basis of 2D-gel patterns. The spots that could be matched with a high degree of confidence and identified as the same protein in both cases (only 39 spots) were selected. Their expression variations were converted into logarithmic values and reported in a scatter plot (Figure 5). This plot clearly shows that the expression variations follow the same trend for 38 spots over 39, demonstrating the similitude between the experimental results and the re-normalized values. In addition the very weak proteomic alteration within the inner membrane, highlighted by the 2D-DIGE comparison on experimentally isolated inner membrane proteomes (1 protein spots), further supports the accuracy of the re-normalization method, which predicted only weak protein expression variation in this compartment (only 0.3% and 1.2% of protein expression variation for LE and HE comparisons respectively).

### Cellular analysis

In order to integrate the results obtained at the mitochondrial level in a global cellular context, we performed a 2D-DIGE analysis on the whole hepatocyte proteome. The comparison was only carried out between WT and HE mice because of the strong dependence of the proteomic response upon TG plasmatic concentration revealed by our mitochondrial analyses. A total of 2736 protein spots were detected in 2D-gels (Additional file 1: Figure S5). Among them, 153 (5.6%) presented a statistically significant (t-test ≤ 0.05) and biologically relevant (+/- 20%: ≤ 0.83 or ≥ 1.2) expression variation, among which 76 could be identified by mass spectrometry (Additional file 8). In opposition to the mitochondrial analysis in which 73% of differentially expressed protein spots could be identified by mass spectrometry, only 50% were identified in the cellular analysis. This observation probably results from the higher complexity of the cellular proteome (2736 detected protein spots vs 1721 in the mitochondrial proteome), therefore inducing slight overlaps of several spots and hampering the proper identification of differentially expressed spots.

### Description of HE mouse metabolic adaptations

Due to the large amount of available data and (sometimes) the high number of spots identified as the same protein (because of post-translational modifications mainly affecting the isoelectric point), only proteins presenting completely up- or down-regulated “spot trains” were considered as biologically up- or down-regulated.

#### Mitochondrial matrix

Several enzymes of glycine and cysteine catabolism are up-regulated in HE mice, indicating that the capacity of these pyruvate-generating pathways is increased, whereas the strong down-regulation of pyruvate carboxylase (which converts pyruvate into oxaloacetate as initial step of gluconeogenesis) suggests that gluconeogenesis presents a decreased capacity. These proteomic changes are probably accountable for more important substrate availability for pyruvate dehydrogenase (PDH), which catalyzes the formation of acetyl-CoA from pyruvate. Accordingly, the PDH enzymatic complex was shown to be up-regulated in HE mice as well as numerous enzymes of β-oxidation and leucine and isoleucine catabolism, indicating that the capacity of these different acetyl-CoA-forming pathways may be increased. Conversely, the down-regulation of one enzyme involved in mitochondrial FFA biosynthesis and three proximal Krebs cycle enzymes (aconitase, isocitrate dehydrogenase, α-ketoglutarate dehydrogenase) suggest that the capacity of these acetyl-CoA-consuming pathways is decreased in HE mice. Altogether, such adaptations may be responsible for a more important acetyl-CoA availability for other metabolic routes.

Some enzymes of valine, proline, arginine and glutamate catabolism were also shown to be up-regulated. Interestingly, the capacity of numerous catabolic (β-oxidation, amino acid degradation) and some anabolic (gluconeogenesis, mitochondrial FFA biosynthesis) pathways seems to be respectively increased and decreased in HE mice. This overall tendency towards catabolism may result in a more important availability of reduced cofactors (NADH, FADH_2_) notably as substrates of the respiratory chain complexes, indirectly leading to a higher ATP production rate. Notably, we found that the two subunits of the electron-transferring flavoprotein (ETFA and ETFB), which acts as an acceptor of electrons from FADH_2_, are strongly up-regulated in HE mice. This protein complex is re-oxidized by the ETF-ubiquinone oxidoreductase, which transfers electrons from ETF to the ubiquinone pool of the respiratory chain.

In addition to aconitase, isocitrate dehydrogenase and α-ketoglutarate dehydrogenase down-regulation, we noticed that three other Krebs cycle enzymes, namely succinyl-CoA dehydrogenase, fumarase and malate dehydrogenase, are strongly up-regulated in HE mice. These proteomic changes suggest that the capacity of the first part of Krebs cycle may be decreased in HE mice, contributing to heighten the availability of acetyl-CoA (as described above), whereas the capacity of the second part of Krebs cycle may be increased. Anaplerosis probably plays a key role in supporting this higher capacity since our results indicate that numerous α-ketoglutarate- (arginine, proline and glutamate catabolism) and succinyl-CoA- (isoleucine and valine catabolism, β-oxidation) producing pathways present an increased capacity in HE mice.

The up-regulation of fumarase and malate dehydrogenase can be rationalized with regard to the up-regulation of ornithine aminotransferase, an urea cycle enzyme, and mitochondrial aspartate aminotransferase. These different enzymes are involved in the “aspartate-argininosuccinate” shunt, which includes both urea and Krebs cycle enzymes and allows recycling of a product of urea cycle (fumarate) into a substrate (aspartate) in order to ensure a continuous operation of the metabolic pathway. Fumarate released by the urea cycle in the cytoplasm is imported to the mitochondrion and is converted into aspartate by three successive enzymatic reactions catalyzed by fumarase, malate dehydrogenase and aspartate aminotransferase. Aspartate is finally exported to the cytoplasm where it can re-enter the urea cycle to form argininosuccinate. Our results suggest that the urea cycle capacity is probably increased in HE mice and that its higher capacity may be supported by an up-regulation of the aspartate-argininosuccinate shunt enzymes.

#### Whole hepatocyte

Only 23 spots corresponding to 12 different mitochondrial proteins were shown to be differentially regulated within the cellular proteome, this low number being attributable to the “dilution” of mitochondrial proteins in terms of diversity and concentration. This dilution effect may lead to an inaccurate quantitation of protein abundance which is inherent to 2D gel-based techniques. For instance, the expression level of some mitochondrial proteins observed at the cellular level correlated with the mitoproteome analysis for 16 spots accounting for 4 different mitochondrial proteins (CPSM, OAT, ACON, SARDH). However, 7 other spots accounting for 7 different mitochondrial proteins (ATPB, IMMT, OTC, AATM, HMCS2, ETFB, SODM) showed an opposite regulation depending on the proteome. These apparent contradictions are unlikely to be of biological origin since all concerned proteins were mostly identified as charge variants (due to post-translational modifications as explained above) within the mitoproteome analysis, whereas only one single spot was detected in the cellular proteome as being differentially expressed. As a consequence, results obtained for these mitochondrial proteins in the context of the cellular proteome comparative analysis were not considered as representative and were not used to infer biological variations.

One glycolytic (α-enolase) and one pentose phosphate pathway (transketolase) enzyme are up-regulated in HE mice compared to WT, suggesting that the capacity of these metabolic routes is increased. Pentose phosphate pathway catabolizes glucose into fructose-6-phosphate and glyceraldehyde-3-phosphate, which can enter glycolysis to produce pyruvate. Consequently, glycolysis and pentose phosphate pathway probably contribute to increase reduced cofactor (NAD(P)H), ATP and pyruvate production rates in HE mice.

Arginase, an urea cycle enzyme, is up-regulated in HE mice whereas cytoplasmic aspartate aminotransferase, which catalyzes the transamination of aspartate into oxaloacetate, is down-regulated, indicating a higher aspartate availability for the urea cycle. Together with the up-regulation of ornithine aminotransferase and aspartate-argininosuccinate shunt enzymes revealed by our mitochondrial analyses, these proteomic changes suggest that the urea cycle capacity may be increased in HE mice. However, this feature remains in doubt since we also noticed that argininosuccinate synthase, another urea cycle enzyme, is down-regulated.

Several cytoplasmic enzymes involved in the defense against oxidative stress, peroxiredoxin 1, glutathione-S-transferases and carbonic anhydrase 3, are strongly up-regulated in HE mice. In case of oxidative stress, molecules (especially unsaturated lipids) are damaged by reactive oxygen species (ROS), generating peroxide chemical groups which prevent the correct achievement of biological functions. Peroxidized molecules must be either reduced or eliminated to allow a continuous operation of cellular metabolism. Peroxiredoxin-1 is involved in the reduction of such molecules [24], whereas glutathione-S-transferases conjugate them with glutathione as a destruction target signal [25]. Regarding carbonic anhydrase 3, previous studies suggested that it may act as an “electron donor” to ROS, indirectly preventing other molecules from oxidative damages, after which it may be targeted to destruction by a specific S-glutathiolation [26].

Ferritin, a protein of iron metabolism, was shown to be up-regulated in HE mice. This protein binds free cytoplasmic Fe^3+^, preventing these highly reactive cations from inducing undesirable chemical effects.

Two proteins involved in cell signaling, ERK2 and NDRG2, are respectively up- and down- regulated in HE mice. ERK2 belongs to the MAPK family and participates to the transduction of cellular proliferation signals. Little is known about NDRG2, but it may be involved in the inhibition of cellular proliferation [27]. Altogether, these results suggest that the hepatocytes of HuApoC-III transgenic mice may be subjected to an induction of cellular proliferation.

## Discussion

Hypertriglyceridemia is commonly associated with diabetes, obesity and metabolic syndrome [1], in which insulin resistance is also a major component of pathogenesis. Since lipid metabolism is under the tight control of insulin action, the discrimination of the relative contribution of HTG and insulin resistance to the pathogenesis of these disorders may be very difficult. Hypertriglyceridemic mice overexpressing the human apolipoproteinC-III have been proven to present no difference of plasma insulin and glucose concentrations [13,16,28], adipocytes insulin response [13], pancreatic insulin secretion [16] and plasma glucose disappearance rates [16] in comparison to WT littermates. Consequently these mice are currently considered as the most adequate model to study the impact of HTG independently of insulin resistance [17]. In the present work, we aimed to characterize the mitochondrial and cellular metabolic adaptations related to HTG in the hepatocytes of the HuApoC-III transgenic mouse by using 2D-DIGE. Since the hepatic lipid physiology of HuApoC-III mice was shown to be strongly dependent on the TG plasmatic concentration [12], comparative proteomic analyses were carried out on both moderately (LE) and severely (HE) hypertriglyceridemic mice. Changes in protein abundance were considered as biologically relevant while exceeding ±20%. Indeed it is well established that 2D-DIGE, in which the use of an internal standard dramatically reduces gel-to-gel experimental variability, enables to detect changes in protein abundance lower than 10% with a p-value inferior to 0.05 with only a small number of replicates [22]. In such a context, ±20% differential expression can be considered as an acceptable cutoff to assess biological variations, as frequently assumed in 2D-DIGE experiments (for example see [29,30]).

In the following sections, the authors will attempt to propose literature-based hypotheses to rationalize their proteomic observations. They insist on emphasizing that these hypotheses must not be considered as firm assertions, but rather aim to provide tracks for future more specific investigations concerning the impact of HTG on liver metabolism.

### The general down- and up-regulation of matricial and inner membrane proteins may be related to several causes in HuApoC-III mouse mitochondria

Considering its primordial function in cellular metabolism, we first aimed to study the impact of HTG on the mitochondrial proteome. Surprisingly, our analyses evidence that matricial and inner membrane proteins are respectively down- and up-regulated in HuApoC-III mice. To our knowledge, such a large-scale phenomenon has never been described in the context of a mitochondrial proteomic analysis. Several possible causes to this phenomenon may be considered in the light of the following previously published findings. HuApoC-III hepatic mitochondria were shown to exhibit a higher respiratory rate in non-phosphorylating condition [18]. This observation has been attributed to the higher activity of the mitochondrial ATP-sensitive K^+^ channel (mitoK_ATP_) in HuApoC-III mice, which results in mild uncoupling of oxidative phosphorylation (OXPHOS) [14]. As observed with other OXPHOS uncoupling mechanisms (e.g. uncoupling proteins, UCPs), the mitoK_ATP_ higher activity has been proposed to lower the ROS production by accelerating the electron transport through the respiratory chain [31]. This has been confirmed in the mitochondria of the HuApoC-III mouse in which lower rates of H_2_O_2_ production were observed, in a reversible manner by mitoK_ATP_ antagonists [20]. Proteomic data from the present matricial analyses further support a lower mitochondrial oxidative stress since peroxide reductase was down-regulated in HuApoC-III mice. On the other hand, a cytoplasmic oxidative stress could be highlighted in these mice as a result of higher activities of xanthine and NADPH oxidases [20]. Therefore, HuApoC-III mitochondria were proposed to be protected from cytoplasmic oxidative stress by the increased activity of mitoK_ATP_ channel [20]. It is worth noticing that abundance changes of mitoK_ATP_ could not be assessed in the present analyses because 2D-DIGE (such as the other gel-based proteomic techniques) does not allow to resolve highly hydrophobic proteins in 2D-gels due to protein aggregation phenomena affecting first- and second-dimension electrophoreses.

Altogether, these findings indicate that the divergence between MAT and MIM proteins may result from one or several of the following causes. (1) Alteration of the mitochondrial morphology: ultrastructural alterations were found in the mitochondria of mice in which HTG was induced by a high-fat diet [32]. These alterations included an increased number of disarrayed cristae in the inner membrane and a reduced electron density in the matrix, which may possibly be rooted to a lower protein content [30-32]. The authors suggested that these alterations may be a consequence of HTG-induced cellular oxidative stress. (2) Lipid peroxidation in the mitochondrial membrane: enhanced cytoplasmic ROS production and subsequent lipid peroxidation byproducts may disrupt the fluidity and integrity of the mitochondrial inner membrane, altering OXPHOS activity and leading to mitochondrial dysfunction. In response to this stress, a membrane biogenesis may be induced through retrograde signaling [33] to compensate the dysfunction of inner membrane proteins. (3) Alteration of mitochondrial protein import: since the import of mitochondrial proteins is partially regulated by cytoplasmic proteins [33], oxidative stress may affect this process and thereby alter the balance between matricial and inner membrane proteins. (4) Alteration of matricial protein folding: our matricial analysis highlighted the up-regulation of the stress-70 protein, which is known to be implicated in the folding of mitochondrial proteins. Since the expression of this protein is inducible through stress-sensitive retrograde signaling [33], its up-regulation may either highlight a defect in the folding of matricial proteins (resulting in the proteolysis of misfold proteins) or further support hypothesis 2. (5) Alteration of the mitochondrial biogenesis signaling: mitochondrial biogenesis has been shown to be directly regulated through cytoplasmic oxidative stress response pathways such as the Nrf2 pathway [34], which may also be implicated in mitoK_ATP_ activity regulation [35]. A complex interaction between these pathways may therefore induce a higher inner membrane biogenesis in HuApoC-III mice.

### The extent of metabolic adaptations occurring in HuApoC-III mouse hepatocytes strongly depends on the TG plasmatic concentration

The 2D-DIGE analysis that we performed on the whole mitoproteome demonstrated that the number and the magnitude of mitochondrial proteomic changes are higher in HE mice compared to LE mice. Furthermore re-normalized and experimental results obtained for sub-mitochondrial compartments revealed that a significant adaptation of the matricial proteome occurs in HE mice but not in LE mice; regarding the inner membrane proteome, surprisingly, it was not significantly modified whatsoever the TG level. These data suggest that the extent of metabolic adaptations occurring in HuApoC-III mouse hepatocytes strongly depends on the TG plasmatic concentration. Accordingly, Aalto-Setälä *et al.* demonstrated that numerous alterations of VLDL and lipid hepatic physiology are more extensive in HE mice compared to LE mice whereas some of them occur only in HE mice [12]. For example, the TG production rate by hepatocytes is twice higher in HE mice compared to control but is not significantly modified in LE mice. Here we demonstrate that the physiological alterations occurring in HuApoC-III mice are probably related to metabolic changes of which the magnitude strongly increases with the hypertriglyceridemia level.

### Re-normalizing protein expression variations can be used as a first approach to counterbalance the effects of a biological large-scale proteomic phenomenon

Global investigation tools such as transcriptomics and proteomics have been developed to study the expression level of genes and proteins in living organisms. Such global approaches suffer from a high influence of experimental artifacts on the quantification of expression levels. Therefore normalization methods had to be designed to correct systematic experimental bias and ensure correct quantification. In 2D-DIGE, such normalization is performed by the DIA (Differential In-gel Analysis) module of the DeCyder software to counterbalance the different fluorescence quantum yields of Cy3 and Cy5 as well as many other artifacts occurring at the level of protein assay, labeling, loading on the gel, etc [36]. In the present work, the comparative analysis of the HuApoC-III mouse mitoproteome revealed a general down- and up-regulation of matricial and inner membrane proteins respectively which hampered the characterization of the different mitochondrial metabolic pathways adaptations to hypertriglyceridemia. As a first approach, we considered this large-scale phenomenon as a biological bias and performed a re-normalization (since the DIA module of DeCyder also performs a normalization, we added the prefix “re-”) aiming to predict the expression variations of proteins inside the two sub-mitochondrial compartments independently from each other. The method that we used here is closely related to the “central tendency normalization”, which is commonly applied in label-free mass spectrometry and transcriptomics and consists in re-centering peptide abundance ratios and intensities of hybridized probes (respectively) around a fixed constant (mean, median, etc.) [37]. For the matricial proteome of HE mice, we could compare the expression variations predicted by the re-normalization to those resulting from an independent 2D-DIGE analysis (Figure 5). This comparison revealed a high similarity between the expression variations obtained by both approaches and confirmed the validity of the re-normalization method.

### In HE mice, the capacity of several metabolic pathways is modified to increase the availability of acetyl-CoA, ATP, glycerol-3-phosphate and NADPH for de novo TG biosynthesis

**Figure 6:**
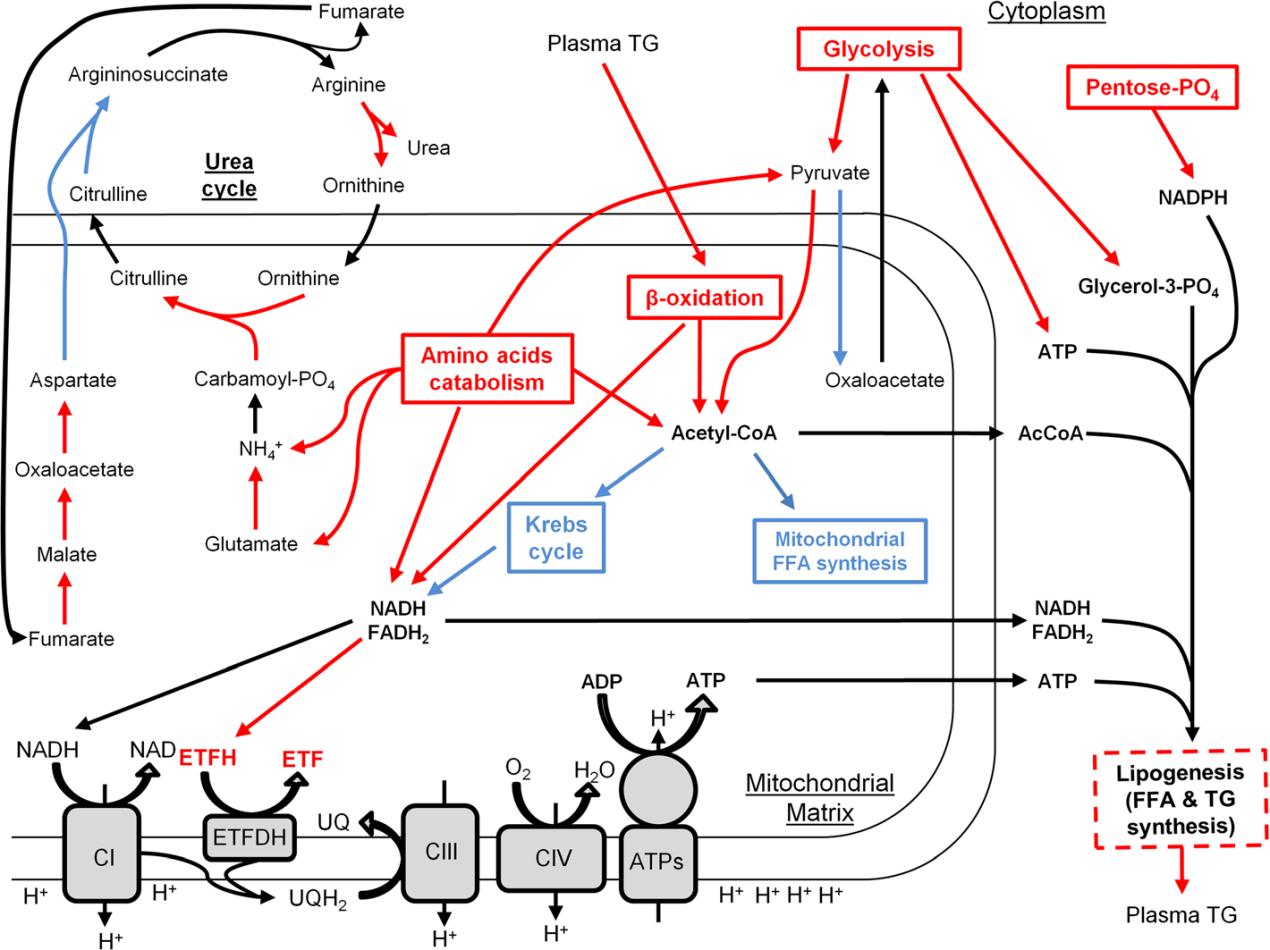
**Main adaptations of the mitochondrial and cellular proteomes in response to HTG in HE mice.** Pathways in red and blue respectively indicate up-regulation or down-regulation as found in our proteomic analyses. Dashed lines indicate an assumed capacity alteration. Stoichiometries of the reactions are not showed. TAG, Triglycerides; CI-III-IV, Complexes I-III-IV; ATPs, ATP Synthase; UQ, Ubiquinone; UQH_2_, Ubiquinol; FFA, Free Fatty Acids, ETFDH, Electron-transferring-flavoprotein dehydrogenase.

The results of our proteomic analyses suggest that the capacities of numerous catabolic (glycolysis, β-oxidation, pentose phosphate pathway, amino acid degradation, pyruvate dehydrogenase) and some anabolic (gluconeogenesis, mitochondrial FFA biosynthesis) pathways are respectively increased and decreased in HE mice (Figure 6). The higher capacity of β-oxidation highlighted here is in good agreement with previous functional measurement showing that liver mitochondria of HuApoC-III mice present a higher state III respiration supported by palmitoylcarnitine which indicates an enhanced β-oxidation rate [20]. We also evidenced that the proximal part of the Krebs cycle exhibits a decreased capacity in HE mice. Altogether these different metabolic alterations may notably be responsible for a higher availability of reduced cofactors (notably NADPH from the pentose phosphate pathway), ATP, acetyl-CoA and some other metabolites (notably glycolytic side-produced metabolites such as glycerol-3-phosphate). The increased availability of reduced cofactors may generate a higher ATP production rate by the respiratory chain contributing to the greater ATP availability. Accordingly, the oxygen consumption rate of liver fragments from HuApoC-III mouse has been shown to be increased compared to control, indicating a higher respiratory activity [14].

Interestingly, Aalto-Setälä *et al.* demonstrated that the hepatic TG production rate is increased in HE mice compared to control, but is not significantly modified in LE mice [12]. In another study, the higher hepatic TG production characterizing the hypertriglyceridemic ldlr^-/-^ mouse has been attributed to an increased *de novo* lipogenesis [38]. Since the substrates of TG biosynthesis are acetyl-CoA, glycerol-3-phosphate, ATP and NADPH, we hypothesize that the metabolic adaptations of HE mouse hepatocytes revealed by our proteomic analyses may sustain an acute cytoplasmic TG biosynthesis, which in turn may support the higher TG production rate characterizing the hepatocytes of HE mice. Since most of the enzymes involved in TG biosynthesis are highly hydrophobic, it is not surprising that 2D-DIGE, which is particularly suitable for the analysis of soluble proteomes, did not allow detecting their probable variation of expression in the present study.

### In HE mice, the capacity of the urea cycle is enhanced to support the higher capacity of amino acid catabolism

The results of our proteomic analyses indicate that several enzymes belonging or associated to the urea cycle are up-regulated in HE mice, suggesting that the capacity of this metabolic pathway is increased (Figure 6). The biological function of urea cycle consists in detoxifying ammonium, mainly produced by amino acid catabolism, by converting it into urea. Since our proteomic analyses demonstrated that the capacity of numerous amino acid catabolic pathways is increased in HE mice, enhanced capacity of urea cycle may serve to prevent hepatocytes from severe damages resulting from ammonium accumulation. Accordingly, it has been shown that mice fed with a high fat diet present an increased urea cycle capacity and activity [39,40]. It has to be emphasized that we also detected that argininosuccinate synthase, a key enzyme of urea cycle, is down-regulated in HE mice so that the overall increase of urea cycle capacity remains in doubt.

### In HE mice, the induction of several anti-ROS systems suggests that the cytosol of hepatocytes is subjected to an important oxidative stress

The 2D-DIGE analysis carried out on the whole hepatocyte proteome revealed that several enzymes involved in the defense against oxidative stress, i.e. peroxiredoxin 1, glutathione-S-transferases (Mu and P classes) and carbonic anhydrase 3, are strongly up-regulated in HE mice. Since anti-oxidant enzymes are substrate-inducible [41], their up-regulation suggests that the cytosol of HuApoC-III mice exhibits an important oxidative stress, implying lipid peroxidation. In agreement with these findings, previous studies demonstrated that HuApoC-III mice exhibit a cytoplasmic oxidative stress originating from higher activities of NADPH and xanthine oxidases whereas a lower ROS production was observed at the mitochondrial level [20]. Consequently, the cytoplasmic oxidative stress was hypothesized to result from intracellular FFA accumulation in hepatocytes. Indeed, such an accumulation may lead to higher activities of peroxisomal and microsomal fat oxidation pathways, which have been shown to induce NADPH and xanthine oxidases and also to be ROS-generating through the activities of cytochromes P450 CYP4A and CYP2E1 [42,43]. As discussed above, the cytoplasmic oxidative stress may induce several ROS-sensitive pathways that may in turn affect mitochondrial functions. Specifically, the ROS-inducible nuclear erythroid 2-related factor (Nrf2) has been shown to be implicated in (1) mitochondrial protection from oxidative stress in mice fed a high fat diet [44], (2) mitochondria biogenesis regulation [34] and (3) probable regulation of the mitoK_ATP_ channel activity [35]. Considering the large-scale mitochondrial adaptations observed here and the evidence of cytoplasmic oxidative stress, the induction of such a cytoplasmic ROS-sensitive pathway seems to be the most reliable hypothesis to explain our mitochondrial results.

### In HE mice, iron over-accumulation may contribute to enhance cytosolic oxidative stress

Recently, hepatic iron overload has attracted much attention because excess iron accumulation causes severe cellular dysfunction by facilitating production of reactive oxygen species, mainly through the Fenton reaction [45]. In this way, hepatic iron overload is newly considered as a predisposing factor and a possible therapeutic target for metabolic syndrome and non-alcoholic steatohepatitis, where hepatic oxidative stress is frequently observed [46]. Interestingly our study evidenced the upregulation of ferritin, involved in free iron storage, in HTG mice liver. As the translation of this protein is known to be enhanced by increased iron levels in hepatocyte cytosol, we can hypothesize that HTG mice undergo a hepatic iron overload [47,48]. Moreover, our proteomic analyses also revealed that urea cycle capacity, as well as hepatocyte proliferation (through up-regulation of ERK2 and down-regulation of NDRG2), are probably enhanced in HE mice. In good agreement with these findings, hepatic iron overload has been shown to induce increased urea cycle capacity and hepatocyte proliferation in mice [49]. Altogether, these results suggest that HTG can be seen as a causative factor for hepatic iron overload, which may participate to the general oxidative stress.

### HuApoC-III mice are more prone to develop non-alcoholic steatohepatitis despite absence of steatosis and insulin resistance

Non-alcoholic fatty liver disease (NAFLD), defined as an alcohol-independent deposit of fat in the liver, includes non-alcoholic steatohepatitis (NASH). NASH is a severe NAFLD form in which fat accumulation is accompanied by necroinflammatory activity, and is now recognized as one of the most common causes of chronic liver disease [50]. Interestingly, our results highlight that the liver of HuApoC-III mice presents a marked oxidative stress probably due to an enhanced extra-mitochondrial FFA oxidation. Oxidative stress is currently considered as a trigger factor for the development of NASH [51]. The present study suggest that neither steatosis (which is another key factor of NASH and is absent in HuApoC-III mice [14,28]) nor insulin resistance and mitochondrial ROS production are necessary for the induction of FFA-mediated oxidative stress. This feature suggests that higher triglyceride and plasma FFA concentrations are sufficient to induce hepatic oxidative stress and therefore increase the probability of NASH development. In good agreement with this assumption, another study demonstrated that HuApoC-III mice fed a high fat diet become insulin-resistant and develop steatosis and NASH [28]. Our results also indicate that iron overaccumulation may participate to the HTG-induced oxidative stress and therefore be implicated in the pathogenesis of NASH. Finally, alterations of the mitochondrial structure (cristalline inclusions made of dysfunctional inner membrane micelles) have been observed in patients with NASH [52]. This may further support the hypothesis of an alteration of the mitochondrial structure in HuApoC-III mice.

## Conclusion

In the present work, we characterized the mitochondrial, sub-mitochondrial and cellular proteomic adaptations related to hypertriglyceridemia in the hepatocytes of the transgenic HuApoC-III mouse model. The mitochondrial analysis demonstrated that the extent of the proteomic response strongly depends on the TG plasma level and revealed a large-scale phenomenon, i.e. a respective general down- and up-regulation of matricial and inner membrane proteins. Further analyses of sub-mitochondrial and cellular proteomes indicate that the metabolic adaptations occurring in HE mouse hepatocytes induce an enhanced acetyl-CoA, glycerol-3-phosphate, ATP and NADPH availability for *de novo* TG biosynthesis. They also strongly suggest that the cytosol of HuApoC-III mouse hepatocytes is subjected to an important oxidative stress, probably as a result of FFA over-accumulation, iron overload and enhanced activity of some ROS-producing catabolic enzymes.

## Materials and methods

### Animals

The human apolipoprotein C-III transgenic mice (line 3707), described elsewhere [14], were bred within the Department of Physiology and Biophysics at the State University of Campinas (Campinas, Brazil) and were provided by Dr. H.C.F. Oliveira. Experiments were approved by the ethics committee of the Universidade Estadual de Campinas and by the Animal Ethical Committee of the University of Liege (Belgium) in accordance with the institutional guidelines for animal care. Mice had access to standard laboratory rodent chow (Nuvital CR1, Parana, Brazil) and water *ad libitum* and were housed at 22 ± 2°C, on a 12 h light/dark cycle. Only males heterozygous apolipoprotein C-III transgenic (HTG, n = 9 comprising LE + HE mice) and nontransgenic (WT, n = 6) littermates, aged from 6 to 7 months, were used in this study. Total plasmatic triglyceride levels were determined by enzymatic-colorimetric method according to the manufacturer’s instructions (Chod-Pap; Roche Diagnostic GmbH, Mannheim, Germany). Mice were divided into three groups according to fasting plasma triglyceride levels: moderately hypertriglyceridemic (LE: 200 mg/dl to 400 mg/dl), severely hypertriglyceridemic (HE: 800 mg/dl to 1000 mg/dl) and control (WT: 40 mg/dl to 80 mg/dl).

### Isolation and purification of mitochondria

Isolations of mitochondria were independently performed on 3 different mice for each TG level group (WT, LE, HE) (total number of samples: 3×3 = 9). Mouse livers were homogenized in 5 ml of sucrose medium (250 mM Sucrose, 1 mM EGTA, 10 mM HEPES-KOH pH 7.4). Cell debris was removed by centrifugation at 600 × g for 10 min at 4°C and mitochondria were finally sedimented from the supernatant by centrifugation at 15 500 × g for 15 min at 4°C. The purification was conducted according to Sickmann *et al*. [53]. Briefly, isolated mitochondria were washed twice in isolation medium, loaded on top of a three step sucrose gradient (3 ml 60%, 4 ml 32%, 3 ml 23%) and centrifuged at 120 000 × g for 1 h at 4°C. The mitochondria were collected from the interface between 32% and 60% sucrose-steps, washed twice with stock solution (250 mM sucrose, 1 mM EDTA, 24 mM Tris-HCl pH 7.4, supplemented with Complete EDTA free protease inhibitor cocktail tablets (Roche Diagnostics)) and stored at -80°C until use.

### Submitochondrial compartment isolation

Isolations of sub-mitochondrial compartments were independently performed using parts of the 3 purified mitochondrial samples for each TG level group (WT, HE) (total number of samples for each sub-mitochondrial compartment: 3×2 = 6). Matrix was isolated according to the method described by Sottocasa *et al.*[54]. Briefly mitochondria were subjected to combined swelling-shrinking followed by sonication. The membrane fraction was pelleted by centrifugation at 105.000 × g during 1 h. The resulting supernatant was finally filtered through an Amicon Ultra-4 PLGC Centrifugal Filter Unit (EMD Millipore) according to manufacturer instructions to raise the protein concentration of the extracts to 5 mg/ml. For mitochondrial inner membrane isolation, an amount of mitochondria corresponding to 1 mg of protein was suspended in 1.5 ml of 10 mM Tris-HCl buffer pH 7.5. After 15 min of incubation at 4°C under agitation, the mitoplasts were pelleted at 16 000 × g during 5 min at 4°C and resuspended in 1 ml of isotonic medium (70 mM sucrose, 210 mM mannitol, 1 mM EDTA, 10 mM Tris-HCl pH 7.5). After 15 min of incubation at 4°C under agitation, mitoplasts were pelleted and resuspended in 230 μl of extraction solution (250 mM KCl, 0.035% Digitonin (w/v), 1 mM EDTA, 30 mM Tris-HCl pH 7.5) and incubated 15 min at 4°C under agitation. Finally, inner mitochondrial membranes were pelleted by Airfuge ultracentrifugation 10 min at 120 000 × g, resuspended in isotonic solution and stored at -80°C until use. All solutions used for the sub-mitochondrial compartment isolations were supplemented with Complete EDTA free (Roche).

### Sample preparation for comparative proteomic studies

Purified mitochondria, matrix and inner membrane extracts were resuspended in a lysis buffer (7 M urea, 2 M thiourea, 2% ASB-14 (w/v), 10 mM DTT, 0.5 M EDTA, Complete EDTA free (Roche), 50 mM Tris-HCl pH 7.5) and intensively vortexed for 30 min at room temperature. After solubilization, the proteins were centrifuged at 10 000 × g to remove any insoluble material. In order to remove excess of salt, fatty acids and nucleic acids, protein extracts were cleaned by using the 2D-Clean Up kit (GE Healthcare).

Extractions of cellular proteomes were independently performed on 3 different mice for each TG level group (WT, HE) (total number of samples: 3×2 = 6). Total cellular proteome was extracted from liver homogenized in sucrose medium supplemented with Complete EDTA free (Roche). In brief, homogenates were solubilized in a 1% SDS solution, were sonicated 30 sec on ice and vortexed 5 min. Insoluble fraction was spun down by centrifugation for 30 min at 50 000 × g. With respect to the complexity of the cellular proteome and the higher presence of contaminating molecules in the total liver protein extracts (in comparison to the mitochondrial extracts), a phenol extraction method has been performed on these samples [55]. In brief, an equal volume of Tris, pH 8.5, buffered phenol (stored at 4°C) was added to 2 mg of protein extracts and vortexed 30 min at 4°C. An equal volume of extraction buffer (10% glycerol (v/v), 2 mM EDTA, 0.7 M sucrose and 50 mM Tris-HCl pH 7.5) was added and the contents were centrifuged 10 min at 15 000 × g. The top phenol phase was transferred to a fresh tube, mixed with 5 volumes of ammonium acetate in methanol (stored at -20°C) and incubated 30 min at -20°C. The mixture were centrifuged at 15 000 × g for 10 min and the supernatants discarded. The pellets were resuspended / centrifuged three times with cold methanol (stored at -20°C) and three times with cold acetone (-20°C). The protein pellets from 2D Clean Up and phenol extraction methods were all finally resuspended in DIGE Label Buffer (7 M urea, 2 M thiourea, 2% ASB-14 (w/v), 10 mM DTT, 0.5 M EDTA, Complete EDTA free (Roche), 50 mM Tris-HCl pH 8.5). Protein concentration was estimated by using RC/DC Protein Assay Kit (BioRad Laboratories) and was adjusted between 5 and 10 mg/mL prior CyDye labeling.

### 2D-DIGE Electrophoresis

All 2D-DIGE experiments were conducted on biological triplicates (3 samples from 3 different mice for each TG level group). Each biological replicate was labeled with both Cy3 and Cy5 (“inversion of labeling”) as a supplementary experimental duplicate to avoid any fluorescence artifact, subsequently generating 2×3 = 6 experimental samples for each condition. For the mitoproteome analysis (3 conditions: WT, LE, HE), 3×(2×3) = 18 experimental samples were thereby generated (9 Cy3- and 9 Cy5-labeled) and had therefore to be dispatched within 9 different 2D gels. For the other analyses (2 conditions: WT, HE), there were 2×(2×3) = 12 experimental samples (6 Cy3 and 6 Cy5-labeled) to be distributed within 6 gels. A Cy2-labeled internal standard (e.g. a pool containing an equal amount of all biological samples) was also loaded on each 2D gel to enable subsequent matching and normalization for inter-gel comparisons, making a total of 18 + 9 = 27 samples for the mitoproteome analysis and 12 + 6 = 18 samples for the other analyses. Twenty five μg of each protein extract were labeled with 0.2 nmole of CyDye (GE Healthcare), for 30 minutes at room temperature. Labeling reactions were stopped by adding 0.5 nmole lysine. Cy2-, Cy3- and Cy5-labelled protein were then pooled together prior to isoelectrofocusing. Pooled samples were reduced by adding 10 mM DTT and then resuspended in the rehydration buffer (7 M Urea, 2 M thiourea, 2% ASB-14, and 0.6% of IPG Buffer (GE Healthcare)) to reach a final volume of 450 μl, and laid on a 24 cm regular strip holder (GE Healthcare). 3-11 NL IPG Drystrips (GE Healthcare) were passively rehydrated in the strip holder for 6 h prior isoelectrofocusing. IEFs were run in the following condition at 20°C: 50 V for 5 h (step), 500 V for 500 Vhr (Step), 1000 V for 800Vhr (Gradient), 8000 V for 13 500Vhr (gradient) and 8000 V for 51 000 Vhr (Step) with a maximum current setting fixed at 50 μA. After isoelectrofocusing, strips were reduced in equilibration buffer (50% glycerol (v/v), 2% SDS (w/v), 6 M urea, 50 mM Tris-HCl, pH 8.8) containing 1% DTT (w/v) for 20 minutes. Then, strips were alkylated in the same buffer containing 2.5% iodoacetamide (w/v) for 20 minutes. After equilibration strips were put on top of a 12.5% acrylamide gel (w/v) for mitochondrial analysis, and on 10% acrylamide gel for cellular analysis, in Laemmli SDS electrophoresis buffer (25 mM Tris, 192 mM Glycine, 1% SDS (w/v)). Electrophoresis was carried out overnight at 1 W/gel (constant power).

### Protein detection, quantitation and statistical analyses

Gels were scanned with the Typhoon 9400 scanner (GE Healthcare) at the wavelengths corresponding to each CyDye. As explained in the previous sub-section, 27 gel images were obtained for the mitoproteome analysis (3 conditions: WT, LE, HE) and 18 for the other analyses (2 conditions: WT and HE). Gels images were analyzed with the DeCyder 7.0 software (GE Healthcare). Briefly, codetection of the three CyDye-labeled forms of each spot was performed using the DIA (Differential In-gel Analysis) module. The DIA module performs the spot detection, the ratio calculation, and the spot abundance normalization via the internal standard. Statistical analyses were carried out in the BVA (Biological Variation Analysis) module after intergel matching. Protein spots that showed a statistically significant Student’s t-test (p ≤ 0.05, n = 6) and ANOVA-1 (p ≤ 0.05, n = 6) for an increase or decrease ranging up to +1.2 or down to -1.2 in normalized ratio intensity were accepted as being differentially expressed between wild-type and HTG mice. In order to ensure relevant quantitation and avoiding any bias due to the extraction methods, Student’s t-test applied to matrix and inner-membrane 2D-DIGE comparisons were considered as significant when ≤ 0.01. Statistical analyses for the re-normalization procedure were performed by using Excel (Microsoft) and JMP 10 (SAS) software.

### In gel digest and mass spectrometry

Matched spots presenting a statistical difference between wild-type and HTG experimental groups were picked using the Ettan Dalt Spot Picker (GE Healthcare). Proteins in gel pieces were subsequently in-gel digested according to Shevchenko *et al.*[56], with some changes. Gel pieces were sequentially washed 3 times with 25 mM NH_4_HCO_3_ and 100% acetonitrile (ACN) to remove excess of detergent and buffer. After the last dehydration in ACN, pieces of gels were rehydrated for 1 h at 4°C with 2 μL of a 5 μg/mL trypsin proteomic grade solution (Roche) diluted in 25 mM NH_4_HCO_3_ in order to ensure sufficient trypsin diffusion and to prevent autocatalysis. Finally, the temperature was raised to 37°C for an overnight digestion.

Peptides were extracted by adding 5 μL of a 1% trifluoroacetic acid (TFA) (v/v)/30% ACN (v/v) solution and vortexed for 30 min. Two μL of the resulting extract was dropped on a 384-600 MTP Anchorchip MALDI target plate (Bruker Daltonics) previously spotted with a 30 mg/mL HCCA matrix (Sigma) solution diluted in acetone. After drop drying, each spot was desalted with cold 10 mM ammonium phosphate solution. Acquisition of mass spectra was carried out using MALDI-TOF/TOF instrumentation (Ultraflex II, Bruker Daltonics) in MS and LIFT MS/MS modes. Mass spectra were analyzed and resulting data were formatted for subsequent database search using the FlexAnalysis software from Bruker Daltonics. MS and MS/MS data were then used for protein identification in the UniProt non-redundant protein database restricted to *Mus musculus* (taxon identifier 10090; 43,537 sequences in the reference proteome set) using MASCOT PMF and MS/MS ion algorithms, respectively, as search engines (http://www.matrixscience.com) with the Bio Tools software (Bruker Daltonics) as interface. For database search, mass error tolerance was fixed at 70 ppm and peptide modifications were assessed as cysteine carbamidomethylation (fixed modification) and methionine oxidation (variable modification). Identified proteins were classified according to their function and sub-cellular or sub-mitochondrial localization on the basis of data reported in UniProtKB/Swiss-Prot and in KEGG Pathway.

## Abbreviations

2D-DIGE: Two dimensional-differential in-gel electrophoresis; ApoC-III: ApolipoproteinC-III; DIA: Differential in-gel analysis; FFA: Free fatty acids; HE: High-expressor; HTG: Hypertriglyceridemia; LE: Low-expressor; LPL: Lipoprotein lipase; OXPHOS: Oxidative Phosphorylation; PSOI: Protein spots of interest; ROS: Reactive Oxygen Species; TG: Triglyceride; TRL: Triglyceride-rich remnants; VLDL: Very-low density lipoproteins; WT: Wild type.

## Competing interests

The authors declare that they have no competing interests.

## Authors’ contributions

Author’s contributions: AEV, HCFO and FES designed study. The mice colony maintenance, plasma triglyceride levels and biological sample provision were performed by AEV and HCFO. Sub-cellular compartment isolations, protein extractions and 2D-DIGE experiments were performed by GE and SG. GE, SG, GM, HCFO, AEV, FF and FES analyzed and discussed data, wrote or revised the manuscript and have primary responsibility for final content. All authors read and approved the final manuscript.

## Supplementary Material

Additional file 1 Supplementary figures and tables. Click here for file

Additional file 2 **Mass spectrometry identification and expression variation of PSOI from mitoproteome comparison.** Protein spots of which the expression level significantly varies in mitochondrial comparisons of HTG LE or HE mice vs control mice (p ≤ 0.05, ± 1.2), classified according to their cellular localization and general function. Protein spots exhibiting biologically significant variation were coloured in blue, protein spots exhibiting statistically significant variation were marked in bold characters. Thirty-four spots exhibited biologically significant variation only in HTG LE vs HTG HE comparison (results not showed) and were coloured in red. Master Number, position of the protein spot in the master gel; Ratio LE or HE, ratio between the normalized volume of the protein spot in control and HTG LE or HE conditions; pI, Isoelectric point; Mw, Molecular Weight; MIM, Mitochondrial Inner Membrane; MAT, Matrix; MOM, Mitochondrial Outer Membrane; IMS, Inter-Membrane Space; ER, Endoplasmic Reticulum; PER, Peroxisome; NUCL, Nucleus; CYTOP, Cytoplasm; CYTOS, Cytoskeleton; EXT, Extra-cellular; UNKN, Unknown. Click here for file

Additional file 3 Supplementary methods. Click here for file

Additional file 4 **Outliers excluded from the re-normalization procedure.** Master Number, position of the protein spot in the master gel; Ratio, ratio between the normalized volume of the protein spot in control and HTG LE or HE conditions; pI, Isoelectric point; Mw, Molecular Weight; MIM, Mitochondrial Inner Membrane; MAT, Matrix. Click here for file

Additional file 5 **Calculation of re-normalized protein spots ratio for matrix and inner membrane proteomes in WT vs LE and HE comparisons.** Results are classified according to proteomes and comparisons. Spots that presented biological relevant variation after renormalization procedure are coloured in blue (ratio ≤ 0.83; down-regulation) or red (ratio ≥ 1.2; up-regulation). Master Number, position of the protein spot in the master gel; Ratio LE or HE, ratio between the normalized volume of the protein spot in control and HTG LE or HE conditions; pI, Isoelectric point; Mw, Molecular Weight. Click here for file

Additional file 6 **Mass spectrometry identification and expression variation of PSOI from mitochondrial matrix proteome comparison.** Protein spots of which the expression level significantly varies in 2D-DIGE comparison of experimentally isolated matrix proteome from HTG HE mice vs control mice (p ≤ 0.01, ± 1.2), classified according to their general function. Master Number, position of the protein spot in the master gel; Ratio, ratio between the normalized volume of the protein spot in control and HTG HE conditions; pI, Isoelectric point; Mw, Molecular Weight. Click here for file

Additional file 7 **Mass spectrometry identification and expression variation of PSOI from mitochondrial inner membrane proteome comparison.** Protein spot of which the expression level significantly varies in 2D-DIGE comparison of experimentally isolated mitochondrial inner-membrane proteome from HTG HE mice vs control mice (p ≤ 0.01, ± 1.2). Master Number, position of the protein spot in the master gel; Ratio, ratio between the normalized volume of the protein spot in control and HTG HE conditions; pI, Isoelectric point; Mw, Molecular Weight. Click here for file

Additional file 8 **Mass spectrometry identification and expression variation of PSOI from cellular proteome comparison.** Protein spots of which the expression level significantly varies in 2D-DIGE comparison of cellular proteome from HTG HE mice vs control mice (p ≤ 0.05, ± 1.2), classified according to their general function. Master Number, position of the protein spot in the master gel; Ratio, ratio between the normalized volume of the protein spot in control and HTG HE conditions; pI, Isoelectric point; Mw, Molecular Weight. Click here for file
