# Supplementary material for: Liver proteomic response to hypertriglyceridemia in human-apolipoprotein C-III transgenic mice at cellular and mitochondrial compartment levels
Source: Lipids Health Dis. 2014 Jul 21;13:116. doi: 10.1186/1476-511X-13-116 (PMC4112841; doi:10.1186/1476-511X-13-116)

Figure S1: Image of the internal standard (Cy2) in the Master gel of mitoproteome 2D-DIGE analysis.

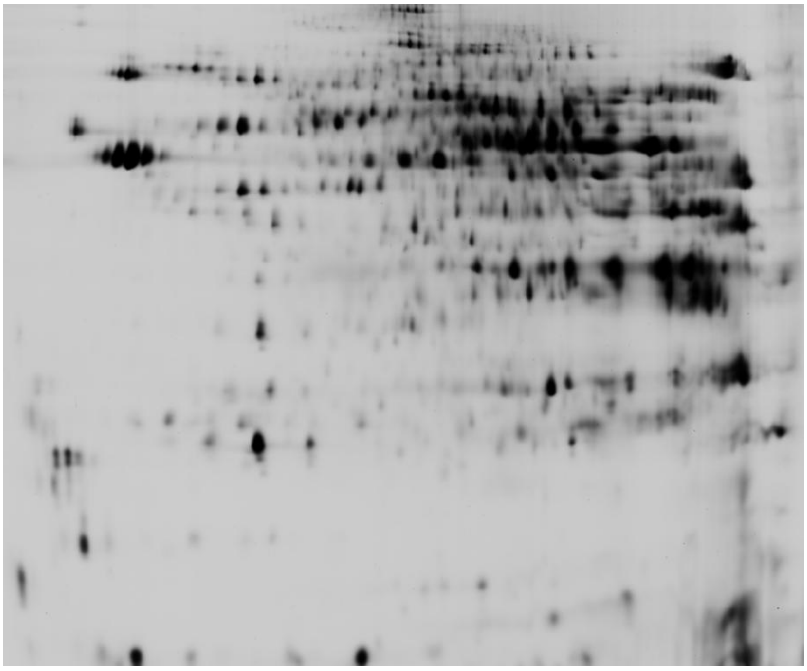

Figure S2: Pie charts classifying the identified PSOI of our mitoproteome 2D-DIGE analysis according to their localization. In Panel A, all identified PSOI are classified following their sub-cellular localization. In Panel B, mitochondrial PSOI are classified following their sub-mitochondrial localization. MITO, mitochondrion; ER, endoplasmic reticulum; PERO, peroxisome; CYTO, cytoplasm; MAT, matrix; IM, inner membrane; IMS, intermembrane space; OM, outer membrane; OTHER, other localization (cytoskeleton, plasma membrane, nucleus or extracellular medium); UNKN, unknown localization.

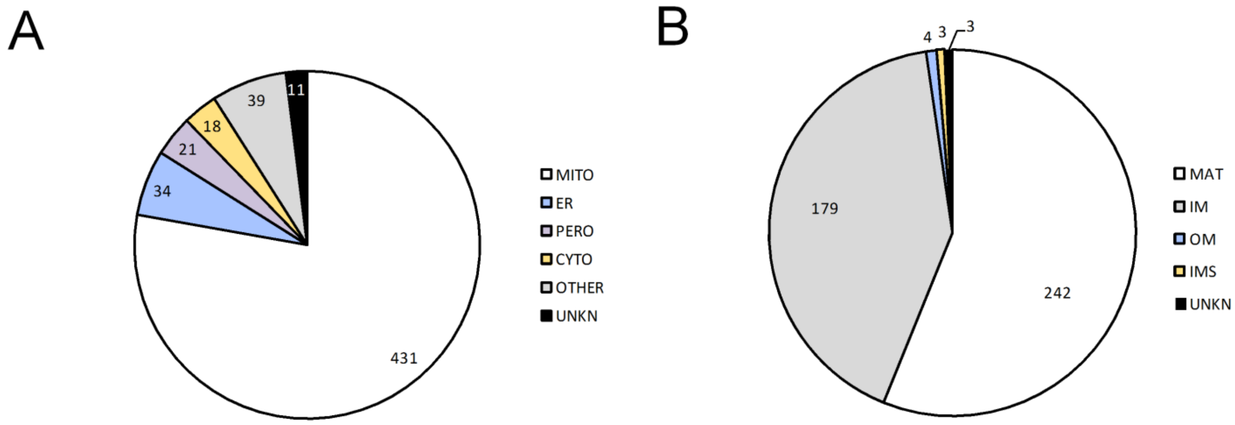

Table S1: Table summarizing the parameters (average: μ and standard deviation: σ) of the normal adjustments performed in Figure 2A. MAT, matrix; IM, inner membrane.

	μ	σ
MAT LE	-0,081822	0,0312873
IM LE	0,0888245	0,0370956
MAT HE	-0,276204	0,1255536
IM HE	0,1498068	0,0607443

Figure S3: Image of the internal standard (Cy2) in the Master gel of matricial 2D-DIGE analysis.

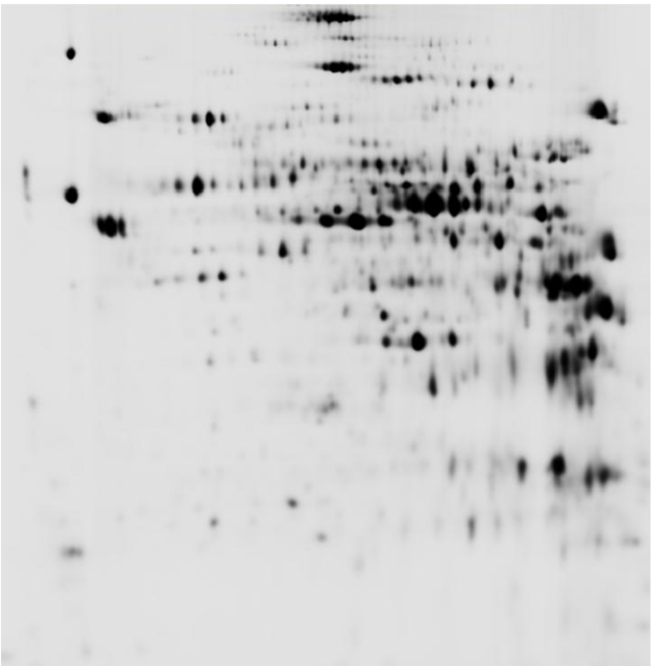

Figure S4: Image of the internal standard (Cy2) in the Master gel of inner membrane 2D-DIGE analysis.

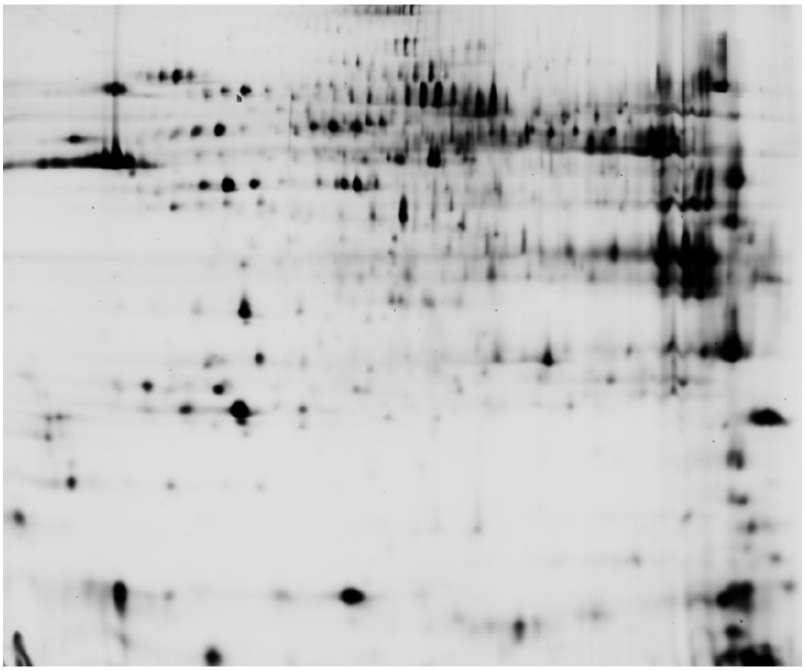

Figure S5: Image of the internal standard (Cy2) in the Master gel of cellular 2D-DIGE analysis.

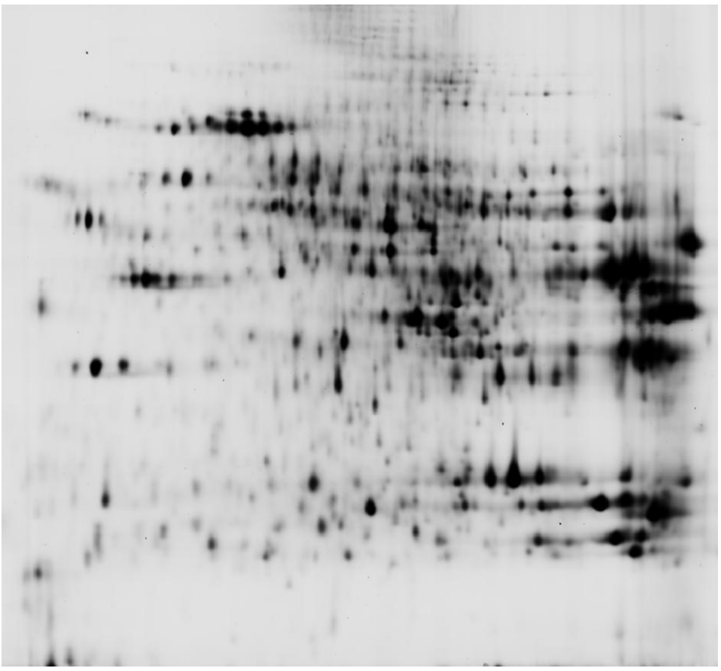

Supplement: Additional file 1 — Supplementary figures and tables. [file 1476-511X-13-116-S1.pdf]
