# Supplementary material for: Liver proteomic response to hypertriglyceridemia in human-apolipoprotein C-III transgenic mice at cellular and mitochondrial compartment levels
Source: Lipids Health Dis. 2014 Jul 21;13:116. doi: 10.1186/1476-511X-13-116 (PMC4112841; doi:10.1186/1476-511X-13-116)
Supplement: Additional file 3 — Supplementary methods. [file 1476-511X-13-116-S3.pdf]

Additional file 3 - Supplementary methods: Detailed re-normalization procedure

Applying a re-normalization requires assuming that the logarithmic forms of PSOI expression variations follow a normal distribution responding to Equation 1:

$$f(x) = \frac{1}{\sigma\sqrt{2\pi}} e^{-\frac{1}{2}\left(\frac{x-\mu}{\sigma}\right)^2} \qquad \text{(Equation1)}$$

where μ is the average and σ the standard deviation of the population. It has to be emphasized that negative and positive logarithmic numbers respectively correspond to *<1* and *>1* expression variations (log 1 = 0). To understand why a logarithmic conversion is necessary, let's consider two expression variations *ax* and *x* related to each other by a factor *a* (*ax/x = a*). The difference between *ax* and *x* logarithmic forms remains constant whatever *ax* and *x* absolute magnitude (*log ax - log x = log (ax/x) = log a = constant*) whereas it is not the case of *ax* and *x* non-logarithmic forms (*ax-x ≠ constant*).

Using JMP, we constructed the distribution charts of PSOI logarithmic expression variations. These graphs (data not shown) revealed that a small number of PSOI exhibited an outlying expression variation and had to be excluded from the analyses to ensure a normal distribution. In case of LE mice, we had to exclude 8 matricial and 6 inner membrane PSOI and, in case of HE mice, 5 matricial and 17 inner membrane PSOI. The proportion of excluded PSOI is low whatever the case (LE matrix: 5.6%, LE inner membrane: 2.1%, HE matrix: 5.2%, HE inner membrane: 10.4%), suggesting that their exclusion will not lead to a significant loss of information. A list of the excluded PSOI is presented in Appendix D.

Figure 2A presents the distribution charts of PSOI logarithmic expression variations obtained after excluding outliers. Each graph was adjusted with a normal curve (Equation 1) of which the parameters (μ and σ) are summarized in Table S1 (Additional file 1). According to the results described in section 2.1, Figure 2A and Table S1 show that μ is respectively negative and positive in matricial and inner membrane distributions and is further from zero in HE mice compared to LE mice. Interestingly, Figure 2A and Table S1 also show that the dispersion of the HE mouse matricial distribution (σ = 0.1256) is twice to fourfold the dispersion of the three other distributions (LE matrix: σ = 0.0313, LE inner membrane: σ = 0.0371, HE inner membrane: σ = 0.0607), indicating that the expression variations of HE mouse matricial PSOI form a more heterogeneous population.

The re-normalization method consists in re-centering the distribution charts of PSOI logarithmic expression variations on the abscissa axis origin. For this purpose, μ was subtracted to the initial logarithmic values, leading to a curve translation along the abscissa axis without modifying σ (Figure 2B). The resulting new logarithmic values were finally re-converted into non-logarithmic ones, corresponding to the re-normalized expression variations.
